# Complete Genome Sequences of Two Streptococcus suis Strains Isolated from Asymptomatic Pigs

**DOI:** 10.1128/MRA.01142-20

**Published:** 2020-11-19

**Authors:** Atsuko Minowa-Nozawa, Takashi Nozawa, Daisuke Takamatsu, Akemi Yoshida, Kazunori Murase, Taisei Kikuchi, Kasumi Ishida-Kuroki, Yoshihiro Nitta, Tsutomu Sekizaki, Ichiro Nakagawa

**Affiliations:** a Department of Microbiology, Graduate School of Medicine, Kyoto University, Kyoto, Japan; b Division of Bacterial and Parasitic Diseases, National Institute of Animal Health, National Agriculture and Food Research Organization, Tsukuba, Ibaraki, Japan; c United Graduate School of Veterinary Sciences, Gifu University, Gifu, Japan; d Laboratory of Genomics, Frontier Science Research Center, University of Miyazaki, Miyazaki, Japan; e Department of Infectious Diseases, Faculty of Medicine, University of Miyazaki, Miyazaki, Japan; f Research Center for Food Safety, Graduate School of Agricultural and Life Sciences, The University of Tokyo, Tokyo, Japan; University of Maryland School of Medicine

## Abstract

Streptococcus suis is an important zoonotic pathogen that causes major economic problems in the pig industry worldwide and serious infections in humans, including meningitis and septicemia. Here, we report the complete genome sequences of two strains isolated from asymptomatic pigs.

## ANNOUNCEMENT

Streptococcus suis is a major swine pathogen that causes various diseases, such as meningitis, septicemia, and endocarditis, which result in large economic losses in the pig industry (1). This bacterium is also an emerging zoonotic pathogen, because life-threatening and fatal S. suis infections have been reported in people engaged in slaughtering pigs and processing raw pork (2, 3). On the other hand, a large proportion of healthy adult pigs have been reported to be asymptomatic carriers of S. suis (4). DAT299 and DAT300, strains of S. suis, are classified as sequence type 114 (ST114) and ST115 in multilocus sequence typing (MLST), respectively, and considered to be weakly pathogenic to pigs and humans (5, 6). Although comparison analysis between pathogenic and weakly pathogenic strains is useful for revelations about the pathogenicity of an organism, whole-genome information of the weakly pathogenic strains is lacking.

Two S. suis strains were isolated using Columbia agar supplemented with sheep blood from tonsils of healthy pigs in a meat inspection center at Okinawa, Japan, and shared by the institutions involved in this study. These strains were incubated for 16 h at 37°C in Todd-Hewitt broth supplemented with 2% yeast extract. The bacterial cells were lysed with lysozyme, and then the genomic DNA was extracted (7). Extracted DNA was short- and long-read sequenced on MiSeq (Illumina) and MinION (Oxford Nanopore Technologies [ONT]) platforms, respectively. The Illumina library was prepared using a Nextera DNA library prep kit, and paired-end reads were generated using MiSeq reagent kit v3 (600 cycles, 2 × 300-bp paired-end reads). A MinION library was prepared from unsheared genomic DNA using a rapid barcoding kit (SQK-RBK004) and sequenced with an R9.4.1 flow cell (FLO-MIN106). Base calling with the high-accuracy model and demultiplexing were performed using Guppy (v2.3.5) (ONT), and the quality control was assessed with MinIONQC (v1.4.2) (8). The ONT adapters were trimmed using Porechop (v0.2.4) (https://github.com/rrwick/Porechop). The Illumina data were preprocessed using Trimmomatic (v0.36) to remove adapter and low-quality sequences (9) and used in a hybrid assembly with MinION long reads using the Unicycler pipeline (v0.4.7b) with default parameters (10). Genome error correction, circularization, and rotation were also implemented in the Unicycler pipeline. The complete genome sequence was then annotated using DFAST (v1.2.6) with default parameters (11). Assembly stats, general genome information, and relevant characteristics are summarized in Table 1.

**TABLE 1:** Assembly stats, general genome information, and relevant characteristics of S. suis strains

Strain and genome information	Data for S. suis strain:	
	DAT299	DAT300
Strain information		
Origin	Healthy pig	Healthy pig
Yr of isolation	2005	2006
ST^*a*^	114	115
Assembly and genome stats		
No. of reads		
MinION (*N*_50_ value [bp])	146,234 (8,789)	363,290 (16,687)
MiSeq	1,029,972	943,120
Total no. of bases		
MinION	274,365,861	1,832,792,270
MiSeq	290,139,123	266,206,331
Coverage (×)	265.34	904.82
Chromosome description^*b*^		
Genome size (bp)	2,127,449	2,319,795
G+C content (%)	41.3	41.1
No. of CDSs^*c*^	2,025	2,207
Avg protein length (bp)	305.4	305.9
Coding ratio (%)	87.2	87.3
No. of rRNAs (*rrn* operon)	12 (4)	12 (4)
No. of tRNAs/tmRNAs^*d*^	56	56
No. of CRISPRs	1	2

a ST, sequence type.

b All genomic stats are output from DFAST pipeline.

c CDSs, coding sequences.

d tmRNAs, transfer-messenger RNAs.

### Data availability.

The complete genome sequences of the two S. suis strains in this study have been deposited in DDBJ/EMBL/GenBank under the accession numbers AP023391 and AP023392. The raw Illumina and MinION read data can be found in the DDBJ Sequence Read Archive with the accession numbers DRR239349 to DRR239350 and DRR239357 to DRR239358, respectively.
